# Multiple interspecies recombination events documented by whole-genome sequencing in multidrug-resistant *Haemophilus influenzae* clinical isolates

**DOI:** 10.1099/acmi.0.000649.v3

**Published:** 2024-02-12

**Authors:** Charlotte Michel, Maria De Los Angeles Argudín, Magali Wautier, Fedoua Echahidi, Benoit Prevost, Olivier Vandenberg, Delphine Martiny, Marie Hallin

**Affiliations:** ^1^​ Department of Microbiology, Laboratoire Hospitalier Universitaire de Bruxelles (LHUB-ULB), Rue Haute 322, 1000 Brussels, Belgium; ^2^​ Belgian National Reference Centre for *Haemophilus influenzae*, Laboratoire Hospitalier Universitaire de Bruxelles (LHUB-ULB), Rue Haute 322, 1000 Brussels, Belgium; ^3^​ Department of Molecular Biology, Cliniques Universitaires Saint Luc (CUSL), Avenue Hippocrate 10, 1200, Brussels, Belgium; ^4^​ Department of Microbiology, Vrije Universiteit Brussel (VUB), Universitair Ziekenhuis Brussel (UZ Brussel), Pleinlaan 2, 1050 Brussels, Belgium; ^5^​ Innovation and Business Development Unit, Laboratoire Hospitalier Universitaire de Bruxelles (LHUB-ULB), Rue Haute 322, 1000 Brussels, Belgium; ^6^​ Centre for Environmental Health and Occupational Health, School of Public Health, Université Libre de Bruxelles (ULB), Avenue Roosevelt 50, 1050 Brussels, Belgium; ^7^​ Division of Infection and Immunity, Faculty of Medical Sciences, University College London, Gower Street, London, WC1E 6BT, UK; ^8^​ Faculty of Medicine and Pharmacy, Mons University, Chemin du Champ de Mars 37, 7000 Mons, Belgium

**Keywords:** DNA uptake machinery, *Haemophilus influenzae*, multidrug resistant, recombination events, uptake signal sequence, whole-genome sequencing

## Abstract

**Introduction.:**

*Haemophilus influenzae* (*Hi*) was long known as an easy-to-treat bacterium, but increasing resistance against beta-lactams and other critically important antibiotics is now a growing concern. We describe here the whole-genome sequencing (WGS) analysis of three non-typeable *Hi* isolates received in 2018–2019 by the Belgian National Reference Centre (NRC) for *Haemophilus influenzae*, as they presented an unusual multi-resistant profile.

**Methods.:**

All three isolates were sequenced by WGS and mapped to the reference isolate *Hi* Rd KW20. Shorten uptake signal sequences (USSs) known to be associated with homologous recombination were sought in *ftsI, murE* and *murF* genes, and inner partial sequences were compared against the blast nucleotide database to look for similarity with other *Haemophilus* species. Their antimicrobial resistance (AMR) genotype was studied. Core-genome multilocus sequence typing (MLST) was performed on the NTHi database pubMLST to place our isolates in the actual worldwide epidemiology.

**Results.:**

The isolates also harboured interspecies recombination patterns in the *murF-murE-ftsI* region involved in cell wall synthesis. The three isolates were multidrug resistant and two of them were also resistant to amoxicillin–clavulanic acid and showed a reduced susceptibility to meropenem. All three isolates belonged to the MLST clonal complex (CC) 422, and WGS revealed that the three were very similar. They harboured mobile genetic elements (carrying *blaTEM-1B*, *mefA* and *msrD* genes associated with resistance), mutations in *gyrA* and *parC* linked to fluoroquinolone resistance as well as remodelling events in *ompP2* that might be related to lower carbapenem susceptibility.

**Conclusion.:**

The *Hi* evolution towards antimicrobial multiresistance (AMR) is a complex and poorly understood phenomenon, although probably linked to a large degree to the presence of USSs and exchange within the family *Pasteurellaceae*. To better understand the respective roles of clonal expansion, horizontal gene transfers, spontaneous mutations and interspecies genetic rearrangements in shaping *Hi* AMR, both analysis of *Hi* communities over time within individuals and worldwide monitoring of non-typeable *Hi* causing infections should be conducted.

## Data Summary

Sequence data are available on NCBI CP121103, CP121104, CP121105. All data are available on DRYAD: doi:10.5061/dryad.6djh9w15x.

**Impact Statement**

In this article, we used whole-genome sequencing to analyse three non-typeable multidrug-resistant *Haemophilus influenzae* (NTHi) isolates that have experienced recombination of the *ftsI* gene (leading to beta-lactam resistance) and the close genes *murE* and *murF* (also involved in cell wall synthesis) with *Haemophilus parainfluenzae* and *Haemophilus haemolyticus*. Their multidrug-resistant profile is of concern as a risk of treatment failure exists for infected patients. We believe that our results are of interest, especially as *H. influenzae* has recently been added to the World Health Organization (WHO) list of Antimicrobial Resistance (AMR) species of concern. The process of evolution leading to shared resistance profiles in NTHi is not yet well understood and should be examined in greater depth.

## Introduction

*Haemophilus influenzae* (*Hi*) is a major human opportunistic pathogen. Present in the normal human respiratory and genital mucosal flora, *Hi* can cause invasive infections such as septicaemia, pneumonia or meningitis, as well as non-invasive infections such as otitis and bronchitis. Classically, most invasive diseases are provoked by serotypeable (capsulated) isolates, with serotype *b* being the most virulent [1], while non-capsulated isolates (non-typeable - NT*Hi*) mainly colonize the upper-respiratory tract [2]. However, non-capsulated isolates have progressively become an important cause of invasive infections [2], especially in countries where *Hib* vaccination has been implemented. In Europe, an increase of *Hi* invasive infections has been witnessed in patients over 60 years old [1, 2] and NT*Hi* are now responsible for the majority of invasive infections [1, 3–5].

*Hi* was long known as an easy-to-treat bacterium. Resistance to beta-lactams, when present, was mostly due to the presence of a transferable beta-lactamase (TEM-1; ROB-1), which was inhibited by clavulanic acid. Over the last decade, the appearance of isolates that are beta-lactamase-negative ampicillin-resistant (BLNAR) has been witnessed and linked to substitutions caused by mutations and/or horizontal gene transfer within the transpeptidase region of *ftsI*, the gene coding for penicillin-binding-protein-3 (PBP3) [1, 2, 4]. These substitutions are indexed so that the BLNAR can be classified into six distinct low- and high-level resistance-related genotypes [1, 2, 6].

*Hi* can undertake inter- and intraspecies recombination [1, 3, 4, 7–9]. Indeed, *Hi* possesses a DNA uptake machinery allowing natural transformation and homologous recombination via a recognition system strongly associated with specific 9 bp short DNA sequence motifs (5′-AAGTGCGGT-3′), called uptake signal sequences (USSs), that are frequently and equally distributed throughout the genome [1, 10]. In particular, recombination of the *fts*I gene among BLNAR NT*Hi* and *Haemophilus haemolyticus* (*Hh*) clinical isolates, as observed by Witherden *et al*. [4], raises questions about the role of such recombinations in the emergence of BLNAR-mediated resistance [4, 11]. While no clear correlation between resistance to third-generation cephalosporins (3GC) and specific substitution pattern(s) exists [2], the recombination phenomenon leading to high-level resistance has been postulated to follow a stepwise evolution, with isolates presenting PBP3 substitutions associated with low-level resistance acquiring second- and then third-stage substitutions through recombination events facilitated by the close proximity of a USS [3]. Of note, Hegstad *et al*. [3] further argue that the epidemiological shift from low-level resistance to high-level resistance BLNAR isolates observed in Norway as well as in Japan a decade earlier occurred when the prevalence of low-resistance BLNAR isolates reached the critical epidemiological threshold of 15–18 % prevalence. Indeed, a sequence type (ST) 422 clone of BLNAR with low susceptibility to fluoroquinolones disseminated in Japan between 2017 and 2019, causing invasive infections. It was then reported in Norway and seems fit to travel rapidly worldwide [3, 12].

Finally, and even though resistance to carbapenems remains scarce [13, 14], it has been postulated that the upregulation of the multidrug-efflux pump AcrAB or porins OmpP, combined with PBP3 substitutions, can lead to resistance to both cephalosporins and carbapenems [1, 15, 16].

Unfortunately, resistance against other critically important antibiotics is also on the rise in *Hi*, and beta-lactam-resistant *Hi* presenting co-resistance to up to four non-beta-lactam groups of antibiotics have already been described, increasing concern regarding limited treatment options. Resistance to fluoroquinolones (FQs), linked to mutations in *gyrA* and *parC* (encoding topoisomerases II and IV, respectively), has been described but remains scarce [3]. Resistance to sulfamethoxazole–trimethoprim (SXT), which is more frequent, is either due to mutations of the dihydrofolate reductase (*dfrA*)- or dihydropteroate synthase (*folP*)-encoding genes, or to the acquisition of the *sul* genes [3]. *Hi* can also present a modification of the macrolide targets (50S rRNA and ribosome-binding proteins), leading to high-level azithromycin resistance, and recent studies have also reported that substitutions in the AcrAB pump can further contribute to macrolide resistance and general reduced susceptibility to antibiotics [1].

In summary, as NT*Hi* are an emerging cause of invasive infections, the continuous rise of resistance in its population seems mainly driven by complex and incompletely documented reshufflings in its chromosome [1, 3]. The involvement of the DNA uptake machinery in the development of resistance in the NT*Hi* population should be explored to better understand how to prevent increasing AMR.

We describe here three NT*Hi* isolates that were sent in 2018–2019 to the Belgian National Reference Centre (NRC) for *Haemophilus influenzae* as they presented an unusual multi-resistant profile. The routine *fts*I sequencing technique was performed [6] as BLNAR were suspected, but the results showed that a part of the *fts*I gene was probably recombined with another *Haemophilus* sp. In order to explore a potential recombination event in the *fts*I gene and the genetic determinants of resistance, as well as the genetic relatedness between these isolates, we performed whole-genome sequencing (WGS) on the three isolates.

## Methods

### Isolate collection, identification, serotyping, antimicrobial susceptibility testing and *ftsI* sequencing

Belgian laboratories are obligatorily required to send their *Hi* invasive isolates to the NRC for national surveillance, but they can also send isolates voluntarily for minimal inhibitory concentration (MIC) determination and *fts*I sequencing when resistance patterns are unusual or when BLNAR is suspected. All isolates are received along with a form that gathers epidemiological and clinical data regarding the related patient. Identification was made using matrix-assisted laser desorption/ionization time-of-flight mass spectrometry (MALDI-TOF MS) (Bruker Daltonics, Bremen, Germany). The search for beta-lactamase was performed using the cefinase test (BD BBL, Erembodegem, Belgium). A screening test with a disc of penicillin 1U and MIC determination was performed. MIC determination for ampicillin (AMP), amoxicillin–clavulanic acid (AMC), cefuroxime (CXM), cefotaxime (CFX), meropenem (MEM), ciprofloxacin (CIP), SXT and tetracycline (TET) is performed routinely by the NRC for treatment purposes, but this is also done for erythromycin (ERY) and azithromycin (AZT), as patients with chronic obstructive pulmonary disease (COPD) are often treated with macrolides. The MICs were prospectively determined with gradient diffusion strips (Etest, bioMérieux, Marcy l’Etoile, France) on MH-F agar plates and interpreted according to the European Committee on Antimicrobial Susceptibility Testing (EUCAST) 2018 algorithm and clinical breakpoints [17].

Biotypes were determined by urease, indol and ornithyl decarboxylase tests (Diatabs, Rosco Diagnostica, Taastrup, Denmark) [18]. The serotype of each isolate was determined by both agglutination (BD Difco, Sparks, Maryland, USA) and detection of the *bexA* gene and serotype-specific capsule genes (a, b, c, d, e and f) by real-time PCR [19, 20].

The transpeptidase domain of the *ftsI* gene is routinely sequenced as described by Ubukata *et al*. [6] for isolates showing resistance to AMC, CXM, CFX, CRO or MEM, as well as for isolates showing resistance to the penicillin screening test or resistance to AMP combined with a negative beta-lactamase test. Briefly, a 621 bp fragment is amplified by PCR and sequenced. Sequences are compared to the ‘wild-type’ reference isolate Rd KW20 (ATCC 51907) sequence [National Center for Biotechnology Information (NCBI) L42023.1]. The amino acid substitutions are detected and listed using BioNumerics v7.6.2 (bioMérieux, Marcy-l'Étoile, France).

### Bacterial culture for DNA extraction

Isolates were plated on chocolate agar PolyViteX with addition of NAD and haemin (bioMérieux, Marcy l’Etoile, France), and incubated in an atmosphere with 5 % CO_2_ for 18 h +/−6 h as previously described [2].

### WGS

Genomic DNA extractions were performed on the Maxwell RSC instrument (Promega Corporation, Madison, WI, USA) with the Maxwell RSC Cell DNA purification kit. Genomic DNA was fragmented using the NEBNext Ultra II FS module and 2×250 bp paired-end DNA libraries were built using the KAPA Hyper Plus kit (Kapa Biosystems, Wilmington, MA, USA) and a selection by Pippin Prep. The PCR amplification step was excluded and a 500 ng input of genomic DNA was used. After equimolar pooling, the WGS was performed using a SP-type flow cell with 500 cycli on a Novaseq 6000 sequencer (Illumina, San Diego, CA, USA). A 1 % PhiX control library was included in each sequencing run. Sequence quality was assessed using FastQC v0.11.4 (https://www.bioinformatics.babraham.ac.uk/projects/fastqc/) [21].

### WGS data analysis

The raw data were downloaded on BioNumerics v7.6.2 as previously described [22, 23]. They were trimmed (by exclusion of reads shorter than 35 bp and longer than 251 bp) and assembled *de novo* with a SPAdes algorithm [3, 24]. The three genomes are available on the public NCBI database (accession numbers CP121103, CP121104, CP121105).

Raw reads were also mapped to the *Hi* reference isolate RD KW20 for a whole-genome single-polymorphism (wgSNP) analysis using closed SNP filtering. Several reference genomes were uploaded on BioNumerics for comparative analysis: the globally used RdKw20 *Hi* reference isolate (L42023.1); R2866 (CP002277), as it was used as reference for NT*Hi* in former publications; and 2018-Y40 (AP022867.1), as it belongs to the multilocus sequence typing (MLST) CC422 multidrug-resistant clone that expended in Japan in 2018. The *Hh* M19346 (NZ_CP031243) and the *Haemophilus parainfluenzae* (*Hpara*) NCTC10665 (LR134481.1) were also used as reference isolates, as they are the closest to our isolates for *murF-murE-ftsI* and available on the GenBank database (Table S3, available in the online version of this article).

Acquired resistance genes were searched via the MobileGenetiqueElementFinder (MGEfinder) online software (https://cge.food.dtu.dk/services/MobileElementFinder/), which was developed for monitoring antimicrobial resistance in the USA [25].

Phylogenetic analysis of *ftsI*, *murE* and *murF* genes was performed with BioNumerics based on multiple alignment using Jukes and Cantor and employing the topscore UPGMA statistical method; branch quality was built by boostrap (Fig S1).

Other genes of interest, such as *omp*P1, *omp*P2, *acr*A, *acr*B and *acr*R, were searched in each isolate’s genome with the module for sequence search, using their position in the reference genome L42023.1 on GenBank; they were then translated in amino acid (aa) sequence via BioNumerics v7.6.2 and aligned to look for substitutions compared to the reference *Hi* Rd KW20 (Data S1).

In order to be sure that the isolates of concern were *Hi*, identification to the species level was performed using the ribosomal multilocus sequence typing (rMLST) plugin on the pubMLST website [26].

cgMLST comparisons was performed using the online plugin on pubMLST [2, 26] and a phylogenic tree built with GrapeTree online software using the 417 public complete genomes of NT*Hi* dated from 2018 to 2022. The isolates were selected with the purpose of investigating the worldwide epidemiology of circulating NT*Hi*, in order to look for similar clones of concern. A second analysis was performed using the closest 46 isolates to our 3 isolates, to zoom in on all of the CC-422 present in the database (total *n*=99).

## Results

The two first isolates (NRCH180079, NRCH180136) were isolated 3 months apart in 2018 from sputa of two patients that suffered from bronchiectasis and COPD, both living in the same province. The third one (NRCH190002) was isolated 6 months later, in 2019, from one of the two previous patients but by a different laboratory. The first patient (isolate NRCH180079) was treated with amoxicillin–clavulanic acid and was cured. The first episode of the second patient (NRCH180136) was not treated but the second event, 6 months later, caused by the NRCH190002 isolate, developed under a long-term azithromycin prophylaxis.

The three isolates were confirmed to be NT*Hi* biotype II according to biochemical factors, agglutination and PCR. The beta-lactamase detection test was positive for all isolates. Table 1 details the MIC values obtained for the three isolates: all showed resistance to AMX, CXM, CRO, CTX, CIP, ERY, AZT and SXT. NRCH180136 and NRCH190002 were also resistant to AMC and showed low susceptibility to MEM (Table 1).

**Table 1.** Charcteristics of isolates from the NRCH collection

Isolate	Year	Site	Clinical data	Bio-type	Sero-type	b-L	MLST		Susceptibility profile																					Acquired resistance genes	Resistance-associated mutations†	MGE
							ST	CC	AMP		AMC		CXM		CRO		CTX		MEM		CIP		SXT		TET		ERY	CLI	AZT			
									MIC	SIR	MIC	SIR	MIC	SIR	MIC	SIR	MIC	SIR	MIC	SIR	MIC	SIR	MIC	SIR	MIC	SIR	MIC	MIC	MIC			
NRCH/ 180079	2018	Sputum	COPD	II	NT	+	1902	422	16	R	1	S	2	R	0.25	R	0.25	R	0.032	S	8	R	32	R	0.25	S	>256	8	>256	*blaTEM-1B*, *msrD* (M.L.S.), *mefA* (M.)	*gyr* A (S84L), *par* C (S84I), *fol* P (insertion SFLYN, position 65-69)	PC15-1a Tn6009
NRCH/ 180136	2018	Sputum	COPD	II	NT	+	1902	422	>256	R	8	R	32	R	0.25	R	0.25	R	0.5	I/S*	16	R	32	R	0.25	S	>256	16	>256	*blaTEM-1B*, *msrD* (M.L.S.), *mefA* (M.)	*gyr* A (S84L), *par* C (S84I), *fol* P (insertion SFLYN, position 65-69)	PC15-1a Tn6009
NRCH/ 190002	2019	Sputum	COPD	II	NT	+	1902	422	>256	R	4	R	>256	R	0.25	R	0.25	R	0.5	I/S*	32	R	32	R	0.25	S	>256	16	>256	*blaTEM-1B*, *msrD* (M.L.S.), *mefA* (M.)	*gyr* A (S84L), *par* C (S84I), *fol* P (insertion SFLYN, position 65-69)	PC15-1a Tn6009

*Meningitis/standard breakpoints.

**Fig. 1.**
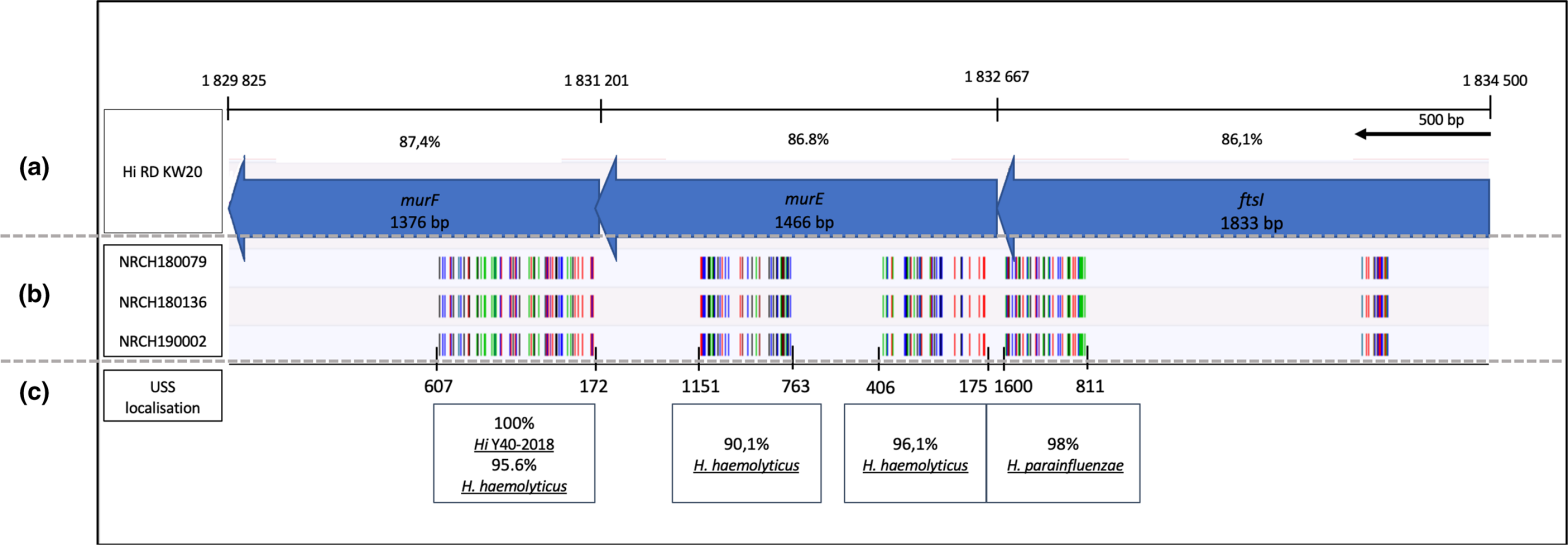
Schematic representation of the *murE*-*murF*-*ftsI* region of the three Belgian non-typeable (NT) *Haemophilus influenzae* (*Hi*) isolates compared to the reference genome *Hi* Rd KW20. (a) Graphic representation of the three genes and percentages of similarity compared to *murE-murF-ftsI of Hi* Rd KW20. (b) Positions of single-nucleotide polymorphisms (SNPs) present in the three strains as compared to *Hi* Rd KW20 (colours are randomly assigned by BioNumerics v7.6.2). (c) Positions of the uptake signal sequences (USSs) found in each gene. The boxes indicate the percentage of similarity of the in-between sequences with their closest match on the blast NCBI database. The small area gathering SNPs appearing in the beginning of the *ftsI* gene were not found to be associated with a known USS sequence. *ftsI*, peptidoglycan DD-transpeptidase or penicillin-binding protein 3; *murE*, UDP-N-acetylmuramoyl-L-alanyl-D-glutamate–2,6-diaminopimelate ligase; *murF*, UDP-N-acetylmuramoyl-tripeptide–D-alanyl-D-alanine ligase.

†For ftsI, see Fig. 1.

AMC, amoxicillin-clavulanic acid;; AMP, ampicillin; AZT, azithromycin; b-L, beta-lactamase; CC, clonal complex; cefuroxime, erythromycin; CFX, cefotaxime; CIP, ciprofloxacin; CLI, clindamycin; COPD, chronical obstructive pulmonary disease; CRO, ceftriaxone; CXM, cefuroxime; L., Lincosamides; M., Macrolides; MEM, meropenem; MGE, mobile genetic element; MIC, minimum inhibitory concentration; MLST, multilocus sequence typing; S, Streptogramins; SIR, interpretation of sensibility to antibiotics according to EUCAST 2018; ST, sequence type; SXT, sulfamethoxazole-trimethoprim; TET, tetracycline.

The analysis of the amino acid sequence of the *ftsI* transpeptidase domain did not allow us to classify any of the three isolates in groups described by Ubukuta *et al*. [6]. Several indexed substitutions (M377I, S385T, N526K and S357N) were present, with the last one being known to be associated with CXM resistance [6, 27], but the sequences did not align well with the Rd KW20 reference isolate, showing only 86.14 % identity. Therefore, a recombination event with another *Haemophilus* species was suspected.

The WGS analysis revealed that the three isolates were very similar: after *de novo* assembly their length was 1 991 786 bp (NRCH180079), 1 991 321 bp (NRCH180136) and 1 991 325 bp (NRCH19002). The mean GC% was 40 for NRCH180079 and 39 for both NRCH180136 and NRCH190002. The average length of median contigs was 146 074 bp and the mean coverage Fig. 1 was 830 for NRCH180079, 868 for NRCH180136 and 489 for NRCH190002, showing good sequencing quality. Mapping to reference followed by wgSNPs analysis showed a difference of 2–3 SNPs between them, as they were 14 909 SNPs distant from the reference isolate Rd KW20.

The identification by rMLST on the pubMLST server confirmed a score of 100 % for *Hi*. The ST determined was 1902 for all three isolates (CC 422). Of note, the three isolates were missing the *fucK* gene*,* one of the seven genes analysed for MLST. When further investigated, the full fucose operon was deleted in the three isolates compared to the reference Rd KW20. Although considered to be a housekeeping gene, *fuc*K has previously been reported missing in 2.1–8.8 % of NT*Hi* isolates [28].

When aligned with the reference isolate *Hi* Rd KW20, the *de novo* assembly of the *murF-murE-ftsI* region of the three isolates suggested a hotspot of remodelling (Fig. 1). To explore further the hypothesis of a mosaicism caused by recombination events in this region involved in cell wall synthesis, USSs (GCGG) described as hotspots of recombination for *Haemophilus* species [29] were sought in the sequences of each gene . Five (*ftsI* and *murE*) or six (*murF*) USSs were found, respectively, and the in-between sequences of these USSs were blasted to evaluate the closest match for each (Table S1).

The multiple alignment of the complete sequence (1833 bp) of the *ftsI* genes remained closer to *Hi* RD KW20 (86.14 %) than to *Hh* M19346 (84.18 %) or *Hpara* NCTC10665 (81.33 %), but when the sequence in-between two of the USSs found (in positions 811 and 1600) was challenged against the blast nucleotide database (blastn) [30], the closest matching sequence was *Hpara*, with 98 % similarity (Fig. 1), which was confirmed by phylogenetic analysis using topscore UPGMA.

The alignment of the complete *murE* gene (1467 bp) showed a sequence that was closer to *Hh* M19346 (90.9 %) than to *Hi* Rd KW20 (86.8 %) or to *Hpara* NCTC10665 (38.8 %). When blasting sequences between USSs found in positions 175 and 406, and between USSs found in 763 and 1151, the closest matching for the in-between sequences was *Hh* M19346 with 96,1 and 90,1 % similarity, respectively (Fig. 1).

Interestingly, the *murF* complete sequence (1376 bp) remained close to the two NT*Hi* reference isolates but was slightly closer to 2018-Y40 (95.7 %) than to R2866 (95.2 %). 2018-Y40 is part of an NT*Hi* multidrug-resistant paediatric clone circulating in Japan [12, 31] that belongs to the same MLST CC422 as the three Belgian isolates. The partial inner-USS sequence (172–607 nt) was 96.5 % similar to the *Hh* reference sequence, compared to *Hi* (91,3 %) and *Hpara* (78,1 %) (Fig. 1).

The search for acquired resistance genes led to the finding of the plasmid pC15-1a carrying *bla_TEM1-B_*, in the three isolates, as well as the transposon Tn6009 carrying the *mefA* and *msrD* genes (conferring resistance to macrolides, lincosamides and streptogramins B). The analysis of the *gyrA* and *parC* genes showed mutations leading to substitutions (S84L and S84I, respectively) correlated to fluoroquinolone resistance [32]. The *folP* gene showed diverse mutations, among which an insertion of 5 aa (SFLYN, position 65–69) correlated to trimethoprim resistance [33]. No *sul* (1 or 2) or *dfrA* (1, 5, 7 and 12) genes were found (Table 1). Additionally, the efflux pump AcrAB and its regulator genes (*acrA*, *acrB* and *acrR*) were studied. Our isolates only harboured a single aa deletion (W88) in *acr*R as compared to RdKW20. AcrA and AcrB harboured 4 aa substitutions each.

In order to explore the possible genes involved in the diminished sensitivity of NRCH180136 and NRCH190002 to meropenem, we looked for the allelic differences between them and NRCH180079. Among the seven genes selected, *ompP*2, *omp*P1/*fad*L and *bam*A, all coding for porin or efflux proteins, could have a possible impact on antibiotic susceptibility. Additionally, a large rearrangement of *ompP2* was found, encompassing deletions and substitutions between aa 176 and 246 of the protein as compared to RdKW20. No USSs were found and, when blasted, the sequence was 100 % identical to diverse reference *Hi* sequences in the NCBI databank (Table S1 and S2).

**Fig. 2.**
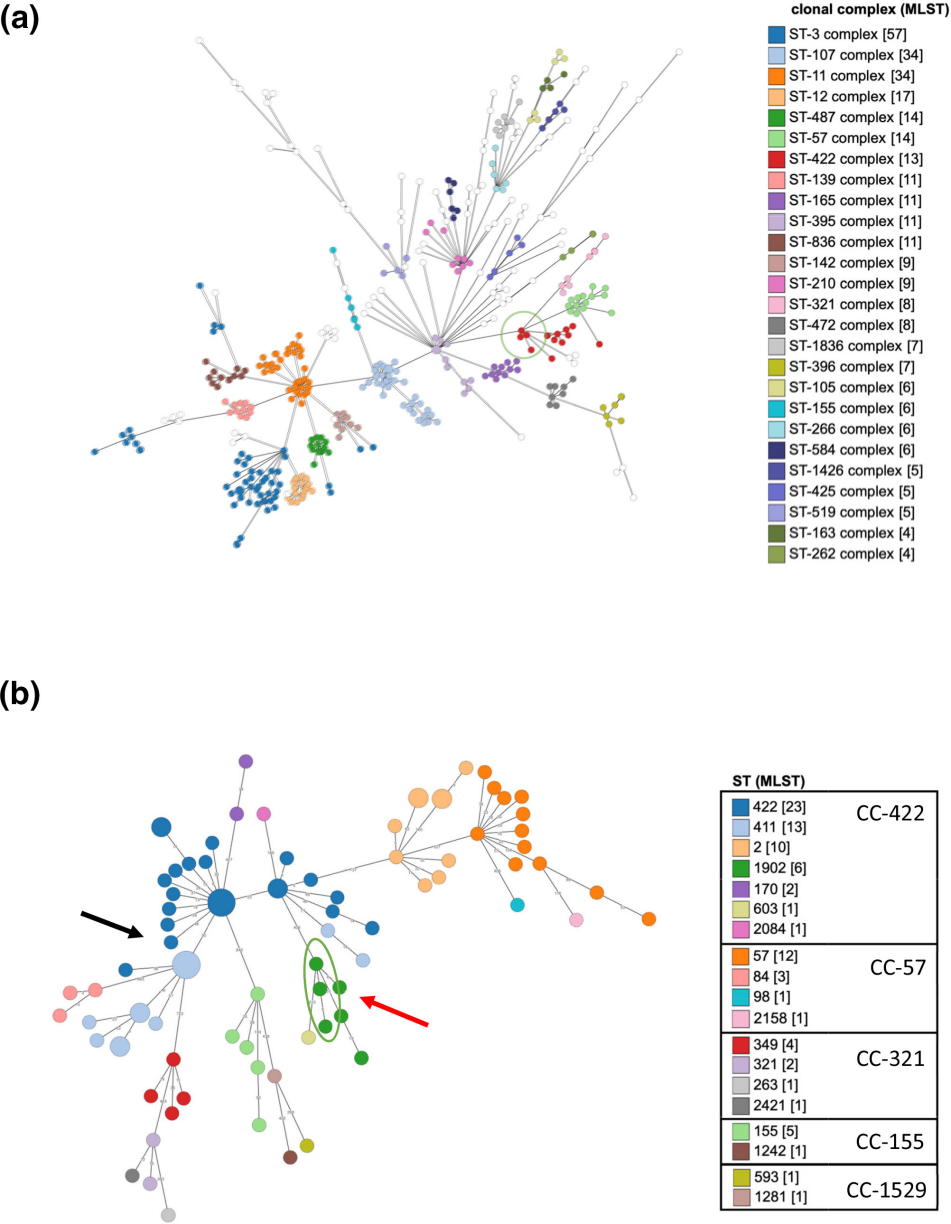
Minimum spanning trees built using the Grapetree plugin on PubMLST based on clonal complex assignment by cgMLST profiling. Length of branches represents the number of allelic differences between sequence types (STs) (nodes). (a) Four hundred and twenty isolates of non-typeable (NT) *Haemophilus influenzae* (*Hi*) are represented. Nodes (STs) are coloured if belonging to a clonal complex (CC) represented by a minimum of four isolates. The three investigated isolates (circled) are clustering with the other CC-422 strains (in red), CC-57 (light green), CC-321 (light pink), CC-84 and CC-1529 (both in white). (b) Zoom-in on the CC-422 and close CCs comprising 99 isolates, including the 3 Belgian isolates. Nodes are coloured following the multilocus sequence typing ST. The isolates of concern, NRCH180079, NRCH180136 and NRCH190002, are circled in green. The NRCH180079 is separated from NRCH180136 and NRCH190002 nodes by 3 and 12 allelic differences, respectively. The black arrow indicates the Japanese isolate 2018-Y40 DNA. The red arrow indicates the isolate 3303, the closest to our isolates, with six allelic difference.

Finally, to situate our isolates in the worldwide NT*Hi* population, we included them in a cgMLST comparison with 417 available sequences of NT*Hi* isolated from 12 different countries, from 2018 to 2022, on the PubMLST site (Fig. 2a). This confirmed the large diversity of the NT*Hi* population, which was distributed in 53 CCs. In total, 13 isolates clustered along with our Belgian isolates in CC422. These came mostly from France, but also the UK, Ireland and Japan.

The cgMLST analysis of the closest 46 isolates to our 3 isolates (Fig. 2b) shows that the 3 cluster together, as they are separated by 3 and 9 allelic differences from each other (maximum length 12). The closest genome found in the database (strain 3303) has six allelic differences and was isolated in France in 2017 as a beta-lactamase-negative ampicillin-sensitive (BLNAS) strain that caused a bacteraemia.

## Discussion

A worldwide emergence of NT*Hi* isolates causing invasive infections has been observed since the introduction of the *Hib* vaccination. In parallel, the recent spread of multidrug-resistant clones of NT*Hi* has led the World Health Organization (WHO) to add *Hi* to its list of AMR species of concern [1]. Increased AMR in *Hi* seems to be evolving among commensal isolates of NT*Hi* [1], catalysed by both the major exposure of children to antibiotics and treatment of chronic infections such as COPD in adults. Indeed, NT*Hi* isolates undergo not only horizontal gene transfers (HGTs) and spontaneous mutations but also undocumented interspecies genetic rearrangements, leading to substitutions in antibiotic targets [1, 3, 4, 7–9].

Here we highlight an example of three isolates showing multidrug-resistant profiles: all three were resistant to AMX, CXM, CFX, CRO, ERY, AZT, CIP and SXT. Two of them also had diminished susceptibility to AMC and MEM and would be of intermediate susceptibility for meningitis treatment. They were isolated from two patients living in the same area. The multidrug resistance profile of one of these isolates led to an exacerbation episode under long-term azithromycin prophylaxis and limited alternative treatment options.

Resistance to macrolides, fluoroquinolones and cotrimoxazole was either related to the acquisition of resistance genes by HGT or to point mutations in target genes. But the deep analysis of both the *ftsI-murE-murF* region and the *ompP2* gene helped us to highlight the complexity of the remodelling that seems to intervene in diverse genes linked to AMR (Table S2 and Data S1). The three isolates studied show several recombination events that occurred with two other commensal *Haemophilus* species (*Hpara* and *Hh*) in the *murF-murE-ftsI* region. Such critical gene rearrangements have already been observed between NT*Hi* and *Hh* for the *ftsI* gene and have also been carried out *in vitro* by co-incubation of NT*Hi* [4, 7]. This might explain why BLNAR are significantly more frequently observed among NT*Hi* than in typeable *Hi* [2]. However, some regions of the genome of typeable *Hi* do show high SNP density and might be hotspots for recombination, suggesting that they are also able to undergo this path of evolution [3, 4, 34]. Another explanation would be that the carriage/infection ratio has evolved to the benefit of NT*Hi* since *Hib* vaccination was deployed [35]. *ftsI* codes for a penicillin-binding protein, and the accumulation of substitutions in the transpeptidase domain are known to modify the resistance pattern to beta-lactams. Our isolates did not fit the groups described by Ubukuta *et al*. [6]. The substitutions observed (M377I, S385T, N526K and S357N) have not been demonstrated to be linked with 3CG resistance and the ones that were described as linked to cephalosporin resistance (L389F, Y557H and G555E) were not found in our isolates [3, 36].

*murF* and *murE* also code for penicillin-binding proteins, which are a UDP-N-acetylmuramoyl-tripeptide–d-alanyl-d-alanine ligase and a UDP-N-acetylmuramoyl-l-alanyl-d-glutamate-2,6-diaminopimelate ligase, respectively. They belong to division/cell wall cluster of genes (*dcw*). The *dcw* region is highly conserved in diverse Gram-negative and Gram-positive bacteria and its regulation depends on a wide panel of regulators and promoters, mostly studied in *Escherichia coli* [37]. Additionally, point mutations of the gene *murE* have once been described as modifying the cell wall and giving a profile of hyper competence in *Hi*, although no other studies have confirmed this point [8].

Hegstad *et al*. studied the acquisition of newly discovered mobile genetic elements (MGEs) in 3CG-resistant isolates [3]. They incriminated an association of mutations occurring in sequences framed by USSs within the transpeptidase domain of *ftsI* and these MGEs [3]. It has been also proven that non-related isolates were evolving to the same mutations set in the *ftsI* gene and that the MGE intake and USS mechanism could be the key to that evolution [29, 31]. In our isolates, none of these MGEs were found and the aligment suggests that the sequences framed by USSs within the transpeptidase domain seem to be inherited from *Hpara*. Similarly*,* sequences framed by USSs in *murE-murF* seem to come from *Hh* (Table 1 and Fig. 1). This phenomenon regarding the *ftsI* gene was documented twice between *Hi* and *Hh*, leading to a heterogenic increase of AMX and CTX MICs [4, 11]. However, this is, to our knowledge, the first report of recombination with *Hpara*.

Thus, homologous recombination by natural transformation seems to be the main mechanism here for the evolution of these central genes’ areas, with USSs being key for exchange between closely related species evolving in the same niche [3, 4, 10, 38]. The key role of this phenomenon is also supported by the fact that the partial sequence of the *ftsI* gene between USSs 229–1600 was more closely related to another oro-pharyngeal commensal, *Aggregatibacter*, when blasted (Table S1). Indeed, USSs comprise ~1 % of the *Hi* genome and are mainly located in coding regions [39]. Furthermore, as the USS-based uptake system is an inheritance of a common ancestor, many USSs are in homologous positions in *Hi* and distant relatives within the family *Pasteurellaceae*, allowing *Hi* to take up DNAs from many other *Haemophilus* species otherwise too divergent to produce recombinants [10]. Watts *et al*. [33] studied AMR in *Haemophilus* sp. colonizing the airways of cystic fibrosis (CF) patients and reported higher colonization and a higher resistance rate for *Hpara* than for *Hi*, with an estimated 15.6 % of patients being persistently colonized for both species [33]. Persistence was linked to AMR with significant differences for AMX, AMC and CTX, leading the authors to conclude that *Hpara* could serve as a reservoir for the emergence of AMR in *Hi* [33]. To obtain a better picture, a global analysis of *Hi*, *Hpara* and *Hh* communities within individual COPD patients and/or children over time should be further conducted.

OmpP2 is the outer-membrane protein found most abundantly in *Hi* [15]. As already described by others, the association between substitutions within *ftsI* and *ompP2* might explain the broad-spectrum-resistant profile observed in our NT*Hi* isolates. Accumulation of mutations in *ompP2* has long been related to the resistance that appears under prolonged beta-lactam treatment in CF patients [40], and studies of *Hi* in chronic rhinosinusitis [41]. NT*Hi* have also shown a capacity for horizontal transfer of *omp*P2 of different patterns in the course of colonization of lung for COPD and in children’s ears [42]. Ineffective OmpP2 mutants can show reduced susceptibility to moxifloxacin and cephalosporins [43]. Mutations in *ompP2* have also been suspected to be involved in the expression of imipenem heteroresistance [15]. In our isolates, *omp*P2 showed remodelling events in the exact same region as Cherkaoui *et al*. [15]. However, carbapenems were never tested for before BLNAR arose and are still only tested for occasionally in our routine diagnostic practice. More evaluation of carbapenem susceptibility should be carried out among NT*Hi* and related to WGS analysis.

Since 2018, Japan has been witnessing the spread of a multidrug-resistant clone in paediatric infections [12, 31]. This clone harbours a BLNAR phenotype and belongs to the same CC422 as the three isolates studied here. The cgMLST minimum spanning tree constructed with NT*Hi* circulating worldwide since 2018 shows our three isolates are not closely related to the 2018-Y40 isolate, representing this clone [12, 31]. By contrast, the closest isolate to our isolates is a French bacteraemic isolate reported as being beta-lactam-susceptible (its *ftsI* was indeed wild-type). This underlines the limits of typing, even cg analysis, to track down resistance transmission in *Hi*.

## Conclusion

Our investigation demonstrates the presence in two Belgian COPD patients of *Hi* isolates that harboured patterns of interspecies recombination with *Hpara* and *Hh* within their *ftsI* and *mur-E-murF* PBP-encoding genes, as well as remodelling events in *ompP2*, letting us draw the hypothesis of a successful resistant clone. Our observations suggest that the accumulation of intra- and inter-species recombination events and point mutations in multiple genes involved in AMR is a potential source of progressive loss of susceptibility to antibiotics in chronic carriage and support the hypothesis that the evolution of NT*Hi* towards AMR follows complex routes, ranging from intra-patient population convergence to inter-patient clonal expansion.

To better understand the respective roles of clonal expansion, horizontal gene transfers, spontaneous mutations and interspecies genetic rearrangements in shaping AMR in *Hi*, both analysis of *Hi* communities over time within individuals and worldwide monitoring of NT*Hi* causing infections should be conducted.

## Supplementary Data

Supplementary material 1
