# Corrigendum: Multiple interspecies recombination events documented by whole-genome sequencing in multidrug-resistant Haemophilus influenzae clinical isolates

**DOI:** 10.1099/acmi.0.000987

**Published:** 2025-01-31

**Authors:** Charlotte Michel, Maria De Los Angeles Argudín, Magali Wautier, Fedoua Echahidi, Benoit Prevost, Olivier Vandenberg, Delphine Martiny, Marie Hallin

**Affiliations:** 1 Department of Microbiology, Laboratoire Hospitalier Universitaire de Bruxelles (LHUB-ULB), Rue Haute 322, 1000 Brussels, Belgium; 2 Belgian National Reference Centre for Haemophilus influenzae, Laboratoire Hospitalier Universitaire de Bruxelles (LHUB-ULB), Rue Haute 322, 1000 Brussels, Belgium; 3 Department of Molecular Biology, Cliniques Universitaires Saint Luc (CUSL), Avenue Hippocrate 10, 1200, Brussels, Belgium; 4 Department of Microbiology, Vrije Universiteit Brussel (VUB), Universitair Ziekenhuis Brussel (UZ Brussel), Pleinlaan 2, 1050 Brussels, Belgium; 5 Innovation and Business Development Unit, Laboratoire Hospitalier Universitaire de Bruxelles (LHUB-ULB), Rue Haute 322, 1000 Brussels, Belgium; 6 Centre for Environmental Health and Occupational Health, School of Public Health, Université Libre de Bruxelles (ULB), Avenue Roosevelt 50, 1050 Brussels, Belgium; 7 Division of Infection and Immunity, Faculty of Medical Sciences, University College London, Gower Street, London, WC1E 6BT, UK; 8 Faculty of Medicine and Pharmacy, Mons University, Chemin du Champ de Mars 37, 7000 Mons, Belgium

In the original article, the number of isolates was misrepresented. The article should have listed 89, not 99 isolates. Additionally, the original article omitted one online resource used to search for acquired resistance genes.

The corrected sentences are:

A second analysis was performed using the closest 46 isolates to our 3 isolates, to zoom in on all of the CC-422 present in the database (total *n*=89).

Figure 2B: (b) Zoom in on the CC-422 and close CCs comprising 89 isolates, including the 3 Belgian isolates.

Previously, the sentences are shown below:

A second analysis was performed using the closest 46 isolates to our 3 isolates, to zoom in on all of the CC-422 present in the database (total *n*=99).

Figure 2B: (b) Zoom in on the CC-422 and close CCs comprising 99 isolates, including the 3 Belgian isolates.

The corrected sentence is:

Acquired resistance genes were searched via the ResFinder online plugin, which was developed for monitoring antimicrobial resistance in the USA [25], and mobile genetic elements were searched via the MobileElementFinder (MGEfinder) online software (https://cge.food.dtu.dk/services/MobileElementFinder/).

Previously, the sentence is shown below:

Acquired resistance genes were searched via the MobileGenetiqueElementFinder (MGEfinder) online software (https://cge.food.dtu.dk/services/MobileElementFinder/), which was developed for monitoring antimicrobial resistance in the USA [25].

