# Supplementary material for: Multiple interspecies recombination events documented by whole-genome sequencing in multidrug-resistant Haemophilus influenzae clinical isolates
Source: Access Microbiol. 2024 Feb 12;6(2):000649.v3. doi: 10.1099/acmi.0.000649.v3 (PMC10928409; doi:10.1099/acmi.0.000649.v3)
Supplement: Supplementary material 1 [file acmi-6-649.v3-s001.pdf]

Supplemental Figure 1: TopScore UPGMA trees of *ftsI (A), murE (B) and murF (C).* All trees were logarithmically scaled by the software. The distances are indicated above each branch.

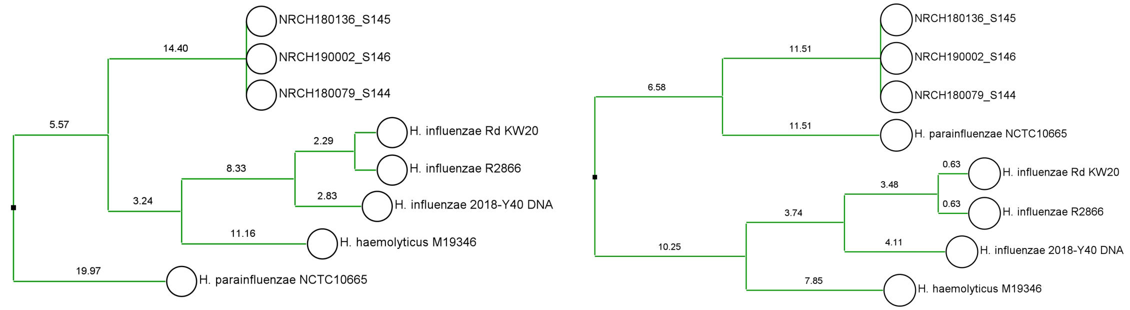

A. TopScore UPGMA trees of *ftsI* complete sequence (left) and sequence between USS nt 1007-1417 (right). The alignment of the complete sequence of complete *ftsI* gene remained closer to *Hi* (86.14%) than to *Hh* (84.18%) or *Hpara* (81.33%). The sequence of *ftsI* between the USS is 98% similar to *Hpara*.

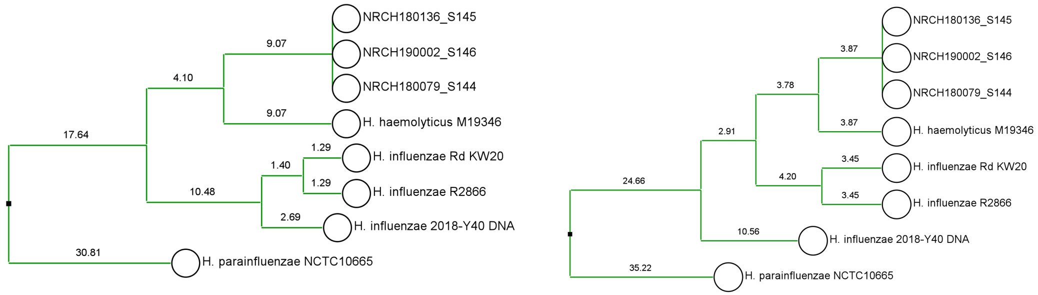

B. TopScore UPGMA trees of *murE* complete sequence (left) and sequence between USS nt 175-766 (right). The complete sequence is closer to *Hh* M19346 (90.9%) and *Hi* Rd KW20-(86.8%) than to *Hpara* NCTC10665 (38.8%). The partial sequence between two USS found in 175 and 766 nt is 94.9% identical to *Hh* M19346 sequence.

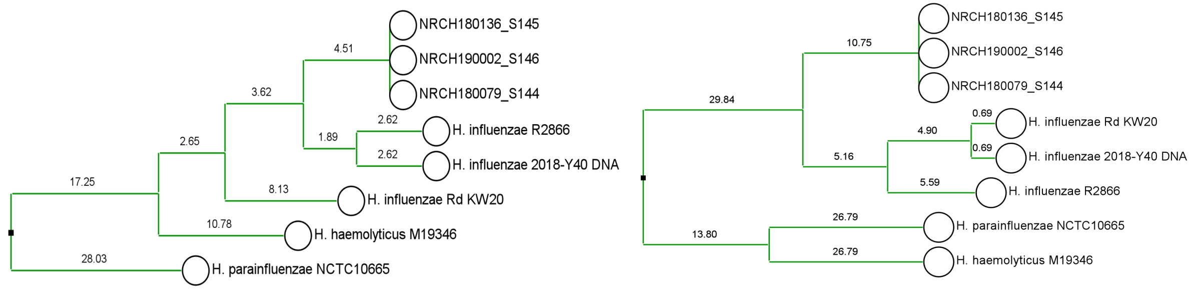

C. TopScore UPGMA trees of *murF* complete sequence (left) and sequence between USS nt 172-607 (right). The first remains closer to the reference strain *Hi* R2866 and *Hi* 2018-Y40 DNA, then *Hi* Rd KW20 (100%) and *Hh* M19346 (91,9%) compared to *Hpara* NCTC 10665 (< 88%). The inner sequence between USS shares 96.5% similarity with the *Hh* reference sequence, compared to *Hi* (91,3%) and *Hpara* (78,1%).

ftsI: peptidoglycan DD-transpeptidase or Penicillin-binding protein 3
murE: UDP-N-acetylmuramoyl-L-alanyl-D-glutamate--2,6-diaminopimelate ligase
murF: UDP-N-acetylmuramoyl-tripeptide--D-alanyl-D-alanine ligase
Hi: Haemophilus influenzae
Hpara: Haemophilus parainfluenzae
Hh: Haemophilus haemolyticus

Gene					
ftsI	USS position on forward strand	in- between sequences	Higher similarity match	closest Hi match	Hh match
	229	229-373	*Hi* 2018- Y40 100%	*Hi* 2018- Y40 100%	/
	373	229-1600	*Aggregatibacter* sp 88%/ *Hpara* 86,1%	84,6%	/
	811	373-811	*Haemophilus* sp 98,6%	82,4%	82,4%
	1207	811-1288	*Hpara* 96,9%	83,0%	/
	1285	811-1600	*Hpara* 98,1%	< 82%	82,5%
	1597	373-1600	*Hpara* 98,1%	< 82%	82,8%
murE	USS position on forward strand	in-between sequences	Higher similarity match	Hi match	Hh match
	175	175 -406	*Hh* 96.1%	*Hi* Rd KW20 92,67%	
	283	283 -406	*Hh* 96%	*Hi* Rd KW20 92,74%	
	403	403 -1151	*Hh* 92.5%	87.2%%	
	763	763 -1151	*Haemophilus* sp 97%	87.4%	91.2%
	1148	1148 -1378	*Haemophilus* sp 99%	/	/
	1378				
murF	USS position on forward strand	in-between sequences	Higher similarity match	Hi match	Hh match
	172	172-607	*Hi* 2018- Y40 100%		95,60%
	187	187 -1189	*Hi* 2018- Y40 100%		< 91%
	604	604 -1189	*Hi* 2018- Y40 100%		< 91%
	1030	1030 -1189	*Hi* 2018- Y40 100%		< 91%
	1189				

Supplementary Table 1: Positions of shorten uptake signal sequences (USS) GCGG found in the genes of the cell wall division region *murF-murE-ftsI* in the isolates of the National Reference Centre Haemophilus collection.

The sequences in-between different combinations of USS were blasted on NCBI Blast nucleotide website with high similarity program selection (megablast).

Hi: Haemophilus influenzae

Hh: Haemophilus haemolyticus

Hpara: Hameophilus parainfluenzae

ftsI: peptidoglycan DD-transpeptidase or Penicillin-binding protein 3

murE: UDP-N-acetylmuramoyl-L-alanyl-D-glutamate--2,6-diaminopimelate ligase

murF: UDP-N-acetylmuramoyl-tripeptide--D-alanyl-D-alanine ligase

Supplementary Table 2: Presence of resistance associated mutations in genes of concern and presence uptake signal sequences (USS) for every isolate of the National Reference centre studied and references genomes.

NRCH180079	Gene	Type of resistance	targted antibiotics	presence of USS (GCGG)	Resistance associated mutations
	ompP2	impermeability	FQ, Macrolides, (Penems?)	No	
	ftsI	modififcation of target	Beta-lactams	Yes	D350N, S357N, S385T
	murE	unknown	unknown	Yes	recombination with Hh M19346
	murF	unknown	unknown	Yes	recombination with Hh M19346
	gyrA	DNA gyrase	Fluoroquinolones	Yes	S84L
	parC	topoisomerase IV	Fluoroquinolones	Yes	S84I
	folP	dihydropteroate synthase	Sulfonamide	Yes	Insertion SFLYN, position 65-69

NRCH180136	Gene	Type of resistance	targted antibiotics	presence of USS (GCGG)	Resistance associated mutations
	ompP2	impermeability	FQ, Macrolides, (Penems?)	No	
	ftsI	modififcation of target	Beta-lactams	Yes	D350N, S357N, S385T
	murE	unknown	unknown	Yes	recombination with Hh M19346
	murF	unknown	unknown	Yes	recombination with Hh M19346
	gyrA	DNA gyrase	Fluoroquinolones	Yes	S84L
	parC	topoisomerase IV	Fluoroquinolones	Yes	S84I
	folP	dihydropteroate synthase	Sulfonamide	Yes	Insertion SFLYN, position 65-69

NRCH19002	Gene	Type of resistance	targted antibiotics	presence of USS (GCGG)	Resistance associated mutations
	ompP2	impermeability	FQ, Macrolides, (Penems?)	No	
	ftsI	modififcation of target	Beta-lactams	Yes	D350N, S357N, S385T
	murE	unknown	unknown	Yes	recombination with Hh M19346
	murF	unknown	unknown	Yes	recombination with Hh M19346
	gyrA	DNA gyrase	Fluoroquinolones	Yes	S84L
	parC	topoisomerase IV	Fluoroquinolones	Yes	S84I
	folP	dihydropteroate synthase	Sulfonamide	Yes	Insertion SFLYN, position 65-69

Hh M19346	Gene	Type of resistance	targted antibiotics	presence of USS (GCGG)	Resistance associated mutations
	ompP2	impermeability	FQ, Macrolides, (Penems?)		no gene ompP2
	ftsI	modififcation of target	Beta-lactams	Yes	D350N
	murE	unknown	unknown	Yes	NA
	murF	unknown	unknown	Yes	NA
	gyrA	DNA gyrase	Fluoroquinolones	Yes	0
	parC	topoisomerase IV	Fluoroquinolones	Yes	0
	folP	dihydropteroate synthase	Sulfonamide	Yes	Insertion SFLYN, position 65-69

Hpara NCTC 10665	Gene	Type of resistance	targted antibiotics	presence of USS (GCGG)	Resistance associated mutations
	ompP2	impermeability	FQ, Macrolides, (Penems?)	Yes	
	ftsI	modififcation of target	Beta-lactams	Yes	D350N
	murE	unknown	unknown	Yes	NA
	murF	unknown	unknown	Yes	NA
	gyrA	DNA gyrase	Fluoroquinolones	Yes	0
	parC	topoisomerase IV	Fluoroquinolones	Yes	0
	folP	dihydropteroate synthase	Sulfonamide	Yes	0

Hi Y40-2018	Gene	Type of resistance	targted antibiotics	presence of USS (GCGG)	Resistance associated mutations
	ompP2	impermeability	FQ, Macrolides, (Penems?)	Yes	
	ftsI	modififcation of target	Beta-lactams	Yes	D350N, S357N, S385T, L389F, V562L
	murE	unknown	unknown	Yes	recombination with Hh M19346
	murF	unknown	unknown	Yes	NA
	gyrA	DNA gyrase	Fluoroquinolones	Yes	S84L
	parC	topoisomerase IV	Fluoroquinolones	Yes	S84I
	folP	dihydropteroate synthase	Sulfonamide	Yes	Insertion SFLYN, position 65-69

Hi R2866	Gene	Type of resistance	targted antibiotics	presence of USS (GCGG)	Resistance associated mutations
	ompP2	impermeability	FQ, Macrolides, (Penems?)	No	
	ftsI	modififcation of target	Beta-lactams	Yes	0
	murE	unknown	unknown	Yes	NA
	murF	unknown	unknown	Yes	NA
	gyrA	DNA gyrase	Fluoroquinolones	Yes	0
	parC	topoisomerase IV	Fluoroquinolones	Yes	0
	folP	dihydropteroate synthase	Sulfonamide	Yes	0

Hi Rd KW20	Gene	Type of resistance	targted antibiotics	presence of USS (GCGG)	Resistance associated mutations
	ompP2	impermeability	FQ, Macrolides, (Penems?)	No	0
	ftsI	modififcation of target	Beta-lactams	Yes	0
	murE	unknown	unknown	Yes	0
	murF	unknown	unknown	Yes	0
	gyrA	DNA gyrase	Fluoroquinolones	Yes	0
	parC	topoisomerase IV	Fluoroquinolones	Yes	0
	folP	dihydropteroate synthase	Sulfonamide	Yes	0

Supplementary Table 3: List of reference strains used

References strains	Serotype	Origin	Country	Date of publishing	Ascession number (NCBI)	MGEfinder
Hi Rd KW20	*d*	unknown	USA	1995	L42023.1	**ISHin1 (transposon)**
Hi R2866	NT	Meningitis and Bacteriaemia	USA	2010	CP002277	blaTEM-1B
Hi Y40-2018	NT	Bacterial pneumonia	Japan	2018	AP022867.1	0
Hpara NCTC 10665	na	Saliva	UK	2018	LR134481.1	0
Hh M19346	na	unknown	USA	2018	NZ_CP031243.1	0
NRCH180079	NT	Sputum	Belgium	2023	CP121103	*Table 1
NRCH180136	NT	Sputum	Belgium	2023	CP121104	*Table 1
NRCH190002	NT	Sputum	Belgium	2023	CP121105	*Table 1

na: not applicable

NT: not typable

Hi: Haemophilus influenzae

Hpara: Haemophilus parainfluenzae

Hh: Haemophilus haemolyticus

MGE: Mobile Genetic Element

MGEfinder: online software

Supplementary Data 1: OmpP2 sequence alignment:
A. Comparison of similarity to *Hi* Rd KW20

Isolate	Similarity of ompP2 to *Hi* Rd KW20 (%)
NRCH180079_S144	97.36
NRCH180136_S145	97.18
NRCH190002_S146	97.18
Hpara NCTC 10665	< 75.0
H. influenzae 2018-Y40 DNA	84.8
Hi R2866	< 86.0

B. Pairwise alignment on ClustalW online software

```
CLUSTAL O(1.2.4) multiple sequence alignment


H._parainfluenzae_NCTC10665      mkktlaalivsavaasaanativydnegtkvelsgslrlilekakaketnqqtgestrta   60
H._influenzae_2018-Y40_DNA       mkktlaaliigalaasaanaavvynnegtkvelggrvsiiaeqstsnrkd------qkhq   54
H._influenzae_R2866              mkktlaalivgafaasaanaavvynnegskvelggrlsviaeqsnntvdd------qkqq   54
H._influenzae_Rd_KW20            mkktlaalivgafaasaanaavvynnegtnvelggrlsiiaeqsnstvdn------qkqq   54
NRCH180079_S144                  mkktlaalivgafaasaanaavvynnegtnvelggrlsviaeqsnstadd------qkqq   54
NRCH180136_S145                  mkktlaalivgafaasaanaavvynnegtnvelggrlsviaeqsnstadd------qkqq   54
NRCH190002_S146                  mkktlaalivgafaasaanaavvynnegtnvelggrlsviaeqsnstadd------qkqq   54
                                 *********:.*.*******::**:***::***.* : :* *::. .  :       :

H._parainfluenzae_NCTC10665      nsalrnagsrfgvkvkhnldndfyalgrlefrfddtds-------rdefgglyakrayvg   113
H._influenzae_2018-Y40_DNA       hgslrnqgsrfnikvthnlgdgyyalgyyetrfinkdidgneknigsgfgsittklayag   114
H._influenzae_R2866              hgalrnqgsrfhikathnfgdgfyaqgyletrfvshyqd-n----adhfddittkyayvt   109
H._influenzae_Rd_KW20            hgalrnqgsrfhikathnfgdgfyaqgyletrfvtkaseng----sdnfgditskyayvt   110
NRCH180079_S144                  hgalrnqgsrfhikathnfgdgfyaqgyletrfvtkaseng----sdnfgdittkyayvt   110
NRCH180136_S145                  hgalrnqgsrfhikathnfgdgfyaqgyletrfvtktseng----sdnfgdittkyayvt   110
NRCH190002_S146                  hgalrnqgsrfhikathnfgdgfyaqgyletrfvtktseng----sdnfgdittkyayvt   110
                                 :.:*** **** :*..**:.:.:** *  * **             . *..: :* **.

H._parainfluenzae_NCTC10665      lgskatgditfgrqvtiaddlnqtndyeyglipkgayiptsgtgvirydykaieglqlsa   173
H._influenzae_2018-Y40_DNA       lgnkelgeatfglqktiadkistaedkeygviekksyiptegn-aiaytykgiegltlga   173
H._influenzae_R2866              lgnkafgevklgraktiadgitsaedkeygvlnnskyirtngn-tvgytfkgidglvlga   168
H._influenzae_Rd_KW20            lgnkafgevklgraktiadgitsaedkeygvlnnsdyiptsgn-tvgytfkgidglvlga   169
NRCH180079_S144                  lgnkafgevklgraktiadgitsaedkeygviknskyiptngn-tigytfkgidglvlga   169
NRCH180136_S145                  lgnkafgevklgraktiadgitsaedkeygviknskyiptngn-tigytfkgidglvlga   169
NRCH190002_S146                  lgnkafgevklgraktiadgitsaedkeygviknskyiptngn-tigytfkgidglvlga   169
                                 **.*  *: .:*   **** :. ::* ***:: :  ** *.*. .: * :*.*:** *.*

H._parainfluenzae_NCTC10665      nynfgqrhnd-----------kgrelkvglknafavgalyeagaldarlayghtnfetna   222
H._influenzae_2018-Y40_DNA       syvfggrnfs-----------dyeitdgkvsnavqvgakydannivagiaygrtnykaqq   222
H._influenzae_R2866              nyllaqardtan------pskegevatqsisngvqvgakydannivagiaygrtnyrkni   222
H._influenzae_Rd_KW20            nyllaqkregakntnkqpndkagevrigeinngiqvgakydandivakiaygrtnykyne   229
NRCH180079_S144                  nyllaqery------------egevnpqkisngvqvgakydanniiagiaygrtnyrent   217
NRCH180136_S145                  nyllaqery------------egevnpqkisngvqvgakydanniiagiaygrtnyrent   217
NRCH190002_S146                  nyllaqery------------ggevnpqkisngvqvgakydanniiagiaygrtnyrent   217
                                 .* :.  .               .     :.*.. *** *:*. : * :***:**:. :
```

```
H._parainfluenzae_NCTC10665     -------ahshrldgflaslgytindfkligd--fgyahdkndna-----------klnk   262
H._influenzae_2018-Y40_DNA      -------aktqqvngalatlgyhfddlglllisldsgyaktknkadk----------hekr   265
H._influenzae_R2866             itpkqdlgrkdqvegvlstlgyhfsdlgllvsldsgyaktkyyeqqqqsnstkprydekr   282
H._influenzae_Rd_KW20           ad-----ehtqqlngvlatlgyrfsdlgllvsldsgyaktknyktk----------hekr   274
NRCH180079_S144                 ig-ipglgkkqqvngalstlgyrfsdlgllvsldsgyaktknykak----------hekr   266
NRCH180136_S145                 ig-ipglgkkqqvngalstlgyrfsdlgllvsldsgyaktknykak----------hekr   266
NRCH190002_S146                 ig-ipglgkkqqvngalstlgyrfsdlgllvsldsgyaktknykak----------hekr   266
                                        :..:::* *::*** :.*: *: .   ***: *               . ::

H._parainfluenzae_NCTC10665     fyvspgfeyqvlptskvygnylyehvkvedsnkakthgfllgadyklhkqvvvflegkya   322
H._influenzae_2018-Y40_DNA      yfvspgfqyelmedtnlygnfkyernsvdqgekvrehavlfgidhklhkqvltyiegaya   325
H._influenzae_R2866             yfvspgfqyelmedtnvygnfkyertssdegkktheqavlfgvdhklhkqvltyiegaya   342
H._influenzae_Rd_KW20           yfvspgfqyelmedtnvygnfkyertsvdqgektreqavlfgvdhklhkqlltyiegaya   334
NRCH180079_S144                 yfvspgfqyelmedtnvygnfkyernsvdqgkktrehavlfgvdhklhkqlltyiegaya   326
NRCH180136_S145                 yfvspgfqyelmedtnvygnfkyernsvdqgkktrehavlfgvdhklhkqlltyiegaya   326
NRCH190002_S146                 yfvspgfqyelmedtnvygnfkyernsvdqgkktrehavlfgvdhklhkqlltyiegaya   326
                                ::*****:*:::  :::***: **: . ::.:*.: :..*:* *:*****::.::** **

H._parainfluenzae_NCTC10665     tvkkytantnggytysakindkaigvgmrvyf       354
H._influenzae_2018-Y40_DNA      rtrtnd-----kgktektekeksvgvglrvyf       352
H._influenzae_R2866             rtktng-----kgkaettgkeksvgvglrvyf       369
H._influenzae_Rd_KW20           rtkttn-----gg--vktekeksvgvglrvyf       359
NRCH180079_S144                 rtrtnd-----kgktektekeksvgvglrvyf       353
NRCH180136_S145                 rtrtnd-----kgktektekeksvgvglrvyf       353
NRCH190002_S146                 rtrtnd-----kgktektekeksvgvglrvyf       353
                                 .:.              . ::*::***:****
```
